# A ghost fence-gap: surprising wildlife usage of an obsolete fence crossing

**DOI:** 10.7717/peerj.5950

**Published:** 2018-11-27

**Authors:** Marc Dupuis-Desormeaux, Timothy N. Kaaria, Mary Mwololo, Zeke Davidson, Suzanne E. MacDonald

**Affiliations:** 1 Department of Biology, York University, Toronto, ON, Canada; 2 Lewa Wildlife Conservancy, Isiolo, Kenya; 3 Marwell Wildlife, Winchester, UK; 4 Department of Psychology, York University, Toronto, ON, Canada

**Keywords:** Mammals, Movement, Conservation, Hyena, Rhinoceros, Fencing, Lion, Path fidelity, Kenya, Fence-gap

## Abstract

Wildlife fencing has become more prevalent throughout Africa, although it has come with a price of increased habitat fragmentation and loss of habitat connectivity. In an effort to increase connectivity, managers of fenced conservancies can place strategic gaps along the fences to allow wildlife access to outside habitat, permitting exploration, dispersal and seasonal migration. Wildlife can become accustomed to certain movement pathways and can show fidelity to these routes over many years, even at the path level. Our study site has three dedicated wildlife crossings (fence-gaps) in its 142 km perimeter fence, and we continuously monitor these fence-gaps with camera-traps. We monitored one fence-gap before and after a 1.49 km fence section was completely removed and 6.8 km was reconfigured to leave only a two-strand electric fence meant to exclude elephant and giraffe, all other species being able to cross under the exclusionary fence. The removal and reconfiguration of the fence effectively rendered this fence-gap (which was left in place structurally) as a “ghost” fence-gap, as wildlife now had many options along the 8.29 km shared border to cross into the neighboring habitat. Although we documented some decline in the number of crossing events at the ghost-gap, surprisingly, 19 months after the total removal of the fence, we continued to document the usage of this crossing location by wildlife including by species that had not been previously detected at this location. We discuss potential drivers of this persistent and counterintuitive behavior as well as management implications.

## Introduction

Fencing wildlife reduces human-wildlife conflicts and protects wildlife within its borders (Hayward et al., 2009; Jori et al., 2011). Fencing for conservation is ubiquitous, hotly debated and has many limitations (Creel et al., 2013; Packer et al., 2013; Watson, 2013; Woodroffe, Hedges & Durant, 2014), notably habitat fragmentation, loss of connectivity and direct mortality (Ferguson & Hanks, 2012; Hoare, 1992) and it can also cause behavioral changes (Whitehouse & Schoeman, 2003). When possible, fencing should be temporary, and conservationists encourage the complete removal of fences (Hoole & Berkes, 2010). However, in densely populated areas, fencing has become a permanent solution to mitigating human-wildlife conflicts even though it is costly to maintain. For example, fencing elephant (*Loxodonta africana*) can be challenging as elephant often break fencing that impedes their migratory routes or in order to raid agricultural crops (Kesch, Bauer & Loveridge, 2015; Kioko et al., 2008; Mutinda et al., 2014). However, fencing elephants can deplete vegetation and also lead to mortality in times of drought (Shrader, Pimm & Van Aarde, 2010).

The creation of fence-gaps in perimeter fencing can create safe pathways for wildlife (particularly for fence-breaking elephant) to migrate in and out of conservancies, which meets both the needs of wildlife and the goals of conservancy management by reducing breakage costs and encouraging connectivity (Dupuis-Desormeaux et al., 2016a). Eventually, other wildlife can adopt these new routes and explore new habitat (Clevenger & Waltho, 2005; Druce, Pretorius & Slotow, 2008; Nyaligu & Weeks, 2013).

**Figure 1:**
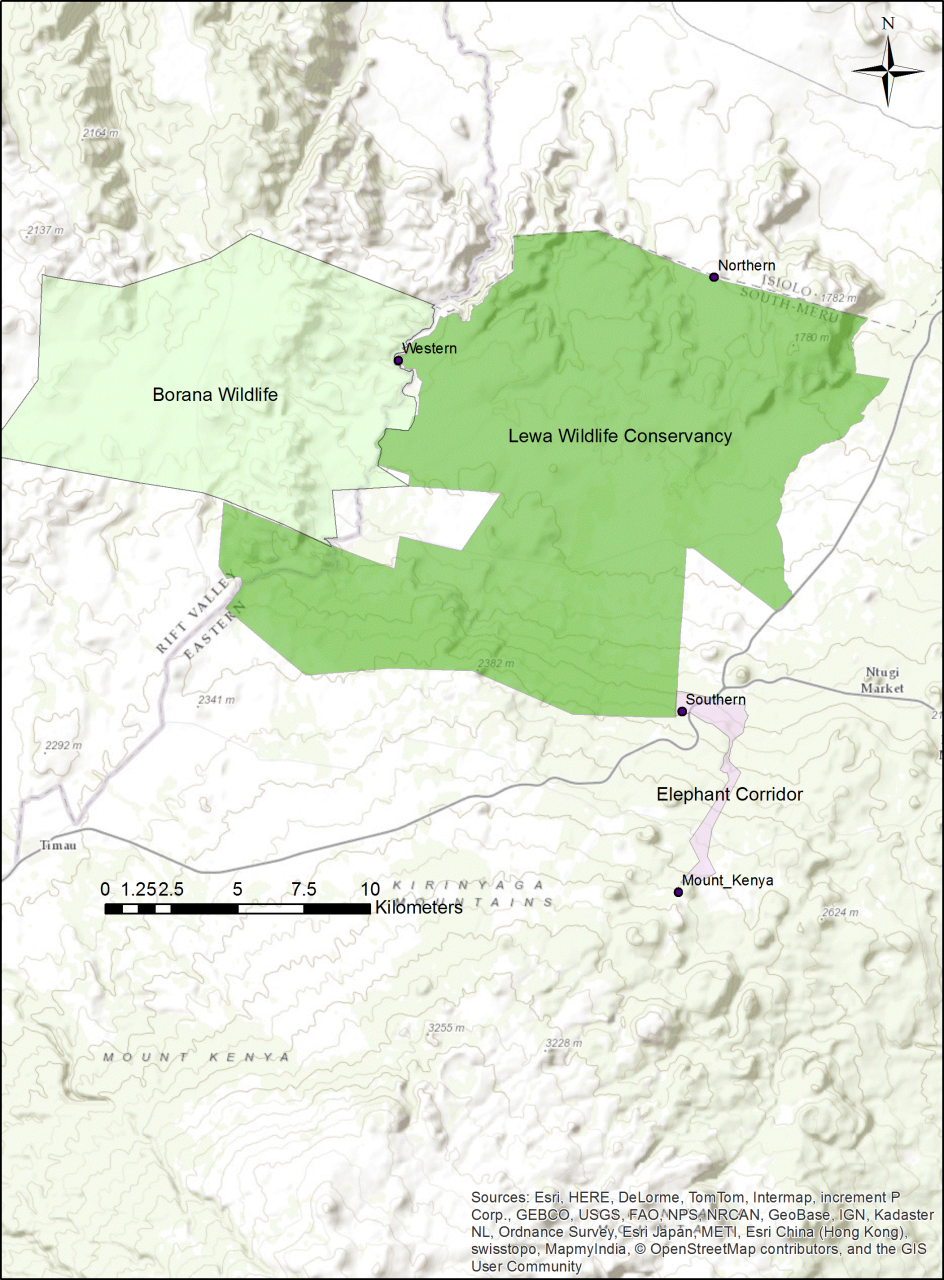
Map of the Lewa–Borana Landscape (LBL). The fence-gaps at the Lewa Wildlife Conservancy are identified on the map.

At our study site in northern Kenya (Fig. 1), elephant and other wildlife have learned the location of three fence-gaps and used them extensively (Dupuis-Desormeaux et al., 2016a). However, fence-gaps create pinch-points in the landscape where wildlife migratory movement is artificially funneled and thus, this could create prey-traps (Ford & Clevenger, 2010). Although, both prey and predator species use the fence-gaps, we have found that predators do not hunt near these fence-gaps, possibly because of the constant elephant traffic (Dupuis-Desormeaux et al., 2015). One of these three fence-gaps, the western gap, connected two adjacent wildlife conservancies (from 2009 to 2015). In 2015, management modified the perimeter fence between the conservancies by making a 6.8 km fence section permeable to most wildlife but excluding elephant and giraffe (*Giraffa camelopardalis reticulata*) and further totally removed a 1.49 km section of fence. Creating this permeable fence or removing the fence completely gave wildlife free movement between the conservancies. Wildlife were no longer restricted to crossing at a predetermined gap in the fence, and thus we expected wildlife to quickly adapt and form new paths that might be better suited to the terrain and the various demands of each species.

The goal of this study was to examine fidelity to a purpose-built crossing structure after that structure had become obsolete, and yet still remained in place. We measured number of individual animal crossings and the number of species using the fence-gap before and after its removal. We predicted that prey animals might quickly abandon the old connecting structure given it was also regularly used by predators. Consequently, we expected to see a significant reduction in both the diversity of species crossing at that point and in the number of individual animals of each species that continued to use that location.

## Materials and Methods

### Study site

**Figure 2:**
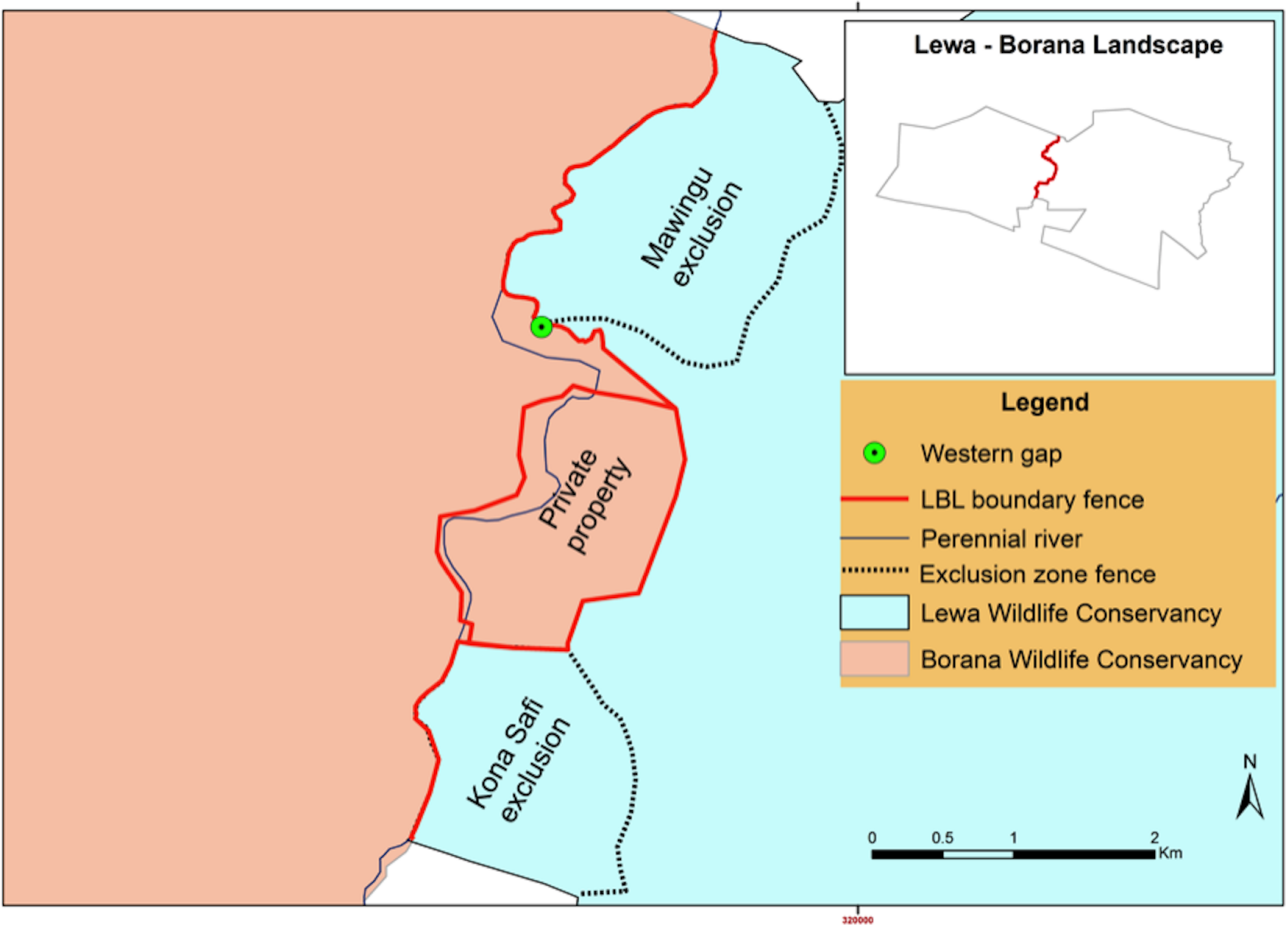
Map of the boundary between the Lewa Wildlife Conservancy and the Borana Conservancy, Isiolo–Meru counties, Kenya. Pre-fence removal (Pre September 2014).

We conducted this study at the border between the 25,000 ha Lewa Wildlife Conservancy in Meru County Kenya (0.20°N, 37.42°E) and the 12,000 ha Borana Wildlife Conservancy (Borana) in Laikipia County Kenya (0.22°N, 37.31°E), together these form the Lewa–Borana landscape (LBL) (Fig. 1). The habitat at LBL consisted of Northern Acacia-Commiphora Bushlands and Thickets with an Afromontane section with significant areas of savannah. Lewa was initially a cattle ranch (1920–1983) and in 1983, in response to declining black rhinoceros (*Diceros bicornis*) population, management converted 2,000 ha to a rhino sanctuary. This sanctuary grew over the subsequent years and in 1995, Lewa officially converted all of its 25,000 ha to a wildlife conservancy and completely fenced its property with a 142 km long, two m high perimeter fence, consisting of 12-strands of alternating live electrical and grounded wires. The perimeter fence was continuous except for a few tended gates and three purpose-built wildlife fence-gaps created to permit safe movement of migratory species in and out of the conservancy. The fence line was monitored continuously for any damage and repaired immediately; therefore animals could only cross through the designated fence-gaps. There was one 30 m-wide fence-gap to the north leading to a pastoralist community, another 20 m-wide fence-gap to the south leading into a 14 km-long purpose-built elephant migratory corridor connected to Mount Kenya and a 20 m-wide fence-gap to the west leading into Borana (Fig. 2). The western border between Lewa and Borana also had two large herbivore exclosures (exclusion zones) on the Lewa side of the perimeter fence, where double-strand electrical wire set at a height of two m limited access to the habitat by elephant and giraffe (the LBL border had a 12-strand fence). Lewa uses exclosures to protect vegetation stands from mega herbivores and to ensure sufficient food supply to both black rhino and Grevy’s zebra (*Equus grevyi*) (Dupuis-Desormeaux et al., 2016b).

**Figure 3:**
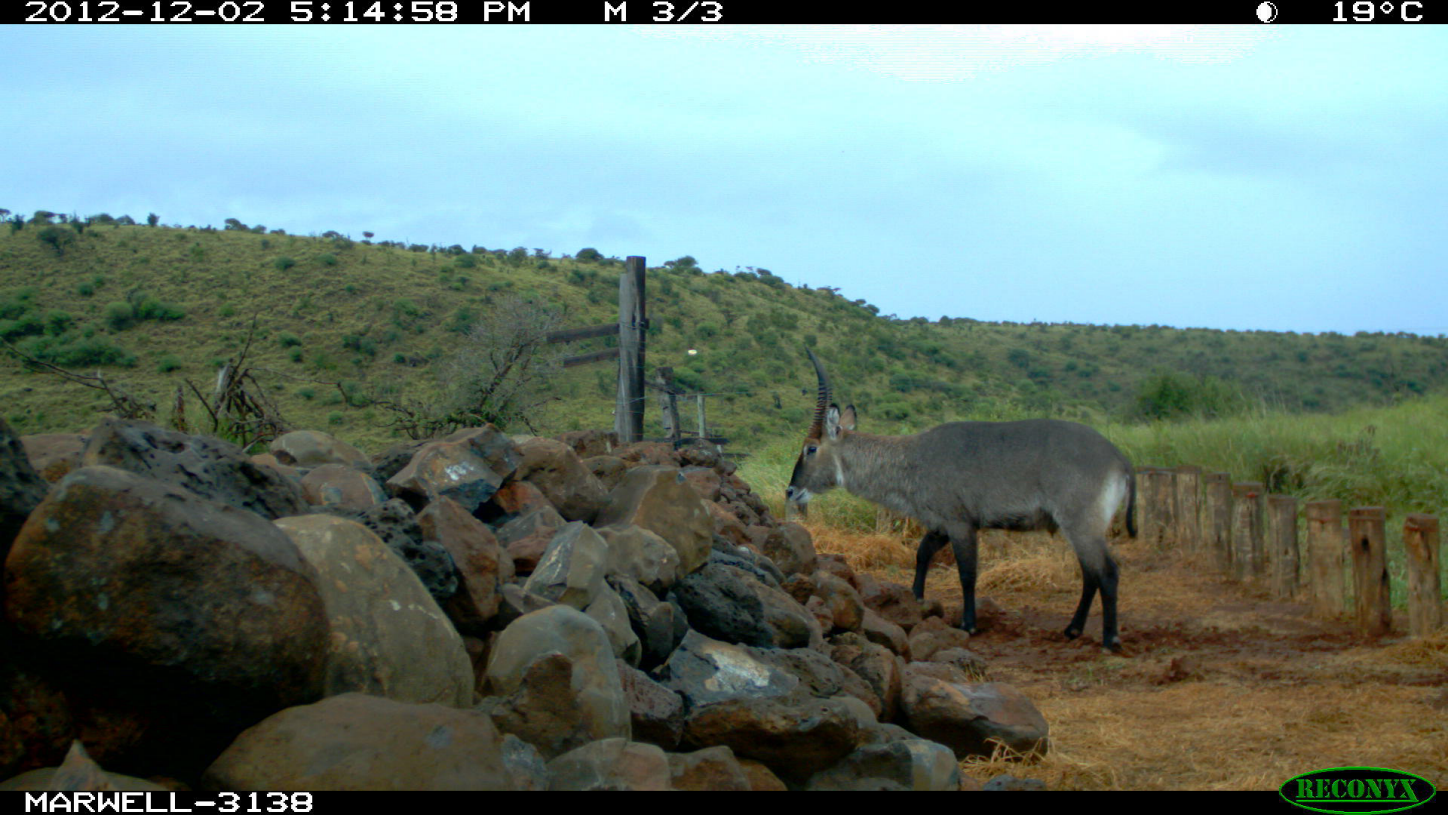
Western fence-gap crossing structure. Note the row of bollards to restrict access of rhinoceros. The electrical fence ends at the rock wall. The camera trap is at the south end of the 20 m wide fence-gap, pointing northwards. Lewa Wildlife Conservancy photo.

The western fence-gap was opened in 2009 at a location of heavy historical fence damage caused by elephant. The fence-gap itself consisted of a sloping wall made of loose volcanic rock built to a height of one m and spanning the width of the 20 m break in the fence wires. In addition, a row of bollards was added directly in front of the rock wall, limiting the ability of rhinoceros to access the fence-gap (see Fig. 3).

**Figure 4:**
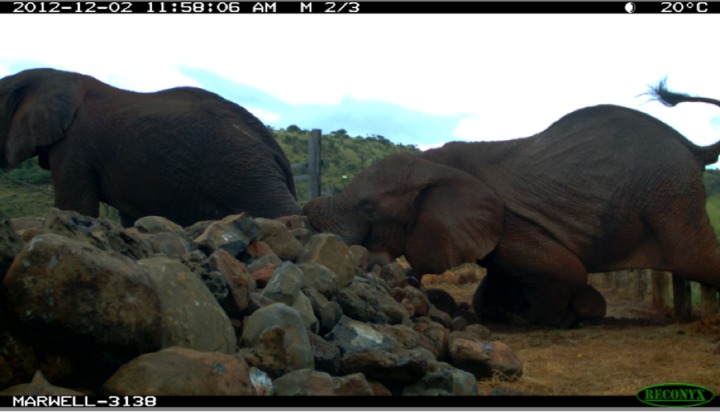
Elephant slipping at the fence-gap between the Lewa Wildlife Conservancy and the Borana Wildlife Conservancy. December 2012. Lewa Wildlife Conservancy photo.

Because of the elevated wall-like feature, this fence-gap was not necessarily easily scalable. For example, we documented several young giraffe approaching and turning back at the fence-gap and elephant tripping over the wet stones (see Fig. 4).

### Monitoring

We monitored the fence-gap using an infrared motion triggered camera (Reconyx HC500 Hyperfire, Holmen, WI, USA). We mounted the cameras in an “elephant-proof” custom built steel housing positioned to be approximately one and half m above the ground and facing across the width of the fence-gap, and protected by electrified deterrent wires. We configured the camera for a three-exposure burst triggered by their inbuilt motion detectors and set the camera for rapid-fire to ensure continuous shooting for as long as the sensor detected movement. Images were stored on secure digital memory cards. Members of the Lewa research department classified the crossing events by recording date, species, number of individuals, and direction of travel. We counted both individual crossings and group crossings. One camera monitors the western fence-gap (pointing northwards), while two cameras monitor the wider northern fence-gap (one pointing eastward and the other pointing westward). The data from the second camera at the northern fence-gap is mostly used as a backup in case of difficulty identifying animals or for counting animals crossing in large groups.

**Figure 5:**
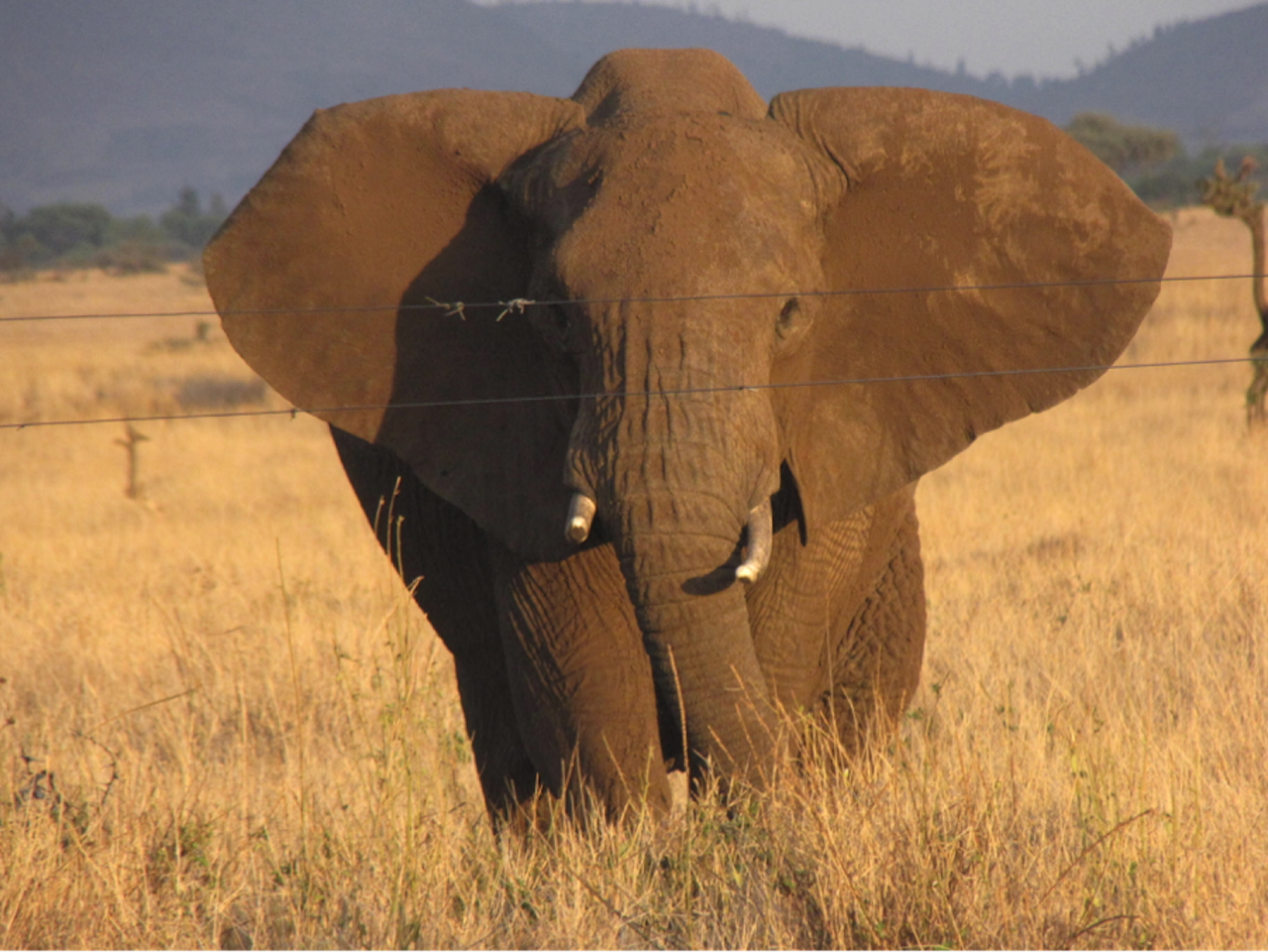
Two-strand electrical fencing installed at a height of two m to exclude elephant and giraffe but permit passage of all other wildlife. Lewa Wildlife Conservancy photo.

In 2015, the management of Lewa and Borana agreed to remove the electrical seven-strand 8.29 km perimeter fence separating their shared border and de facto created a 37,000 ha conservancy. The fence was either removed or modified from October 2014 to September 2015. Phase one (October 2014–January 2015) consisted of modifying the fence by removing the seven-strand fence and replacing it by an electrical two-strand exclusion fence (two m in height) to exclude only giraffe and elephant (see Fig. 5 below). This new exclusionary fence allowed all other species to pass freely below the exclusionary fence to access the two conservancies without the necessity of using the fence-gap.

The fence remained in this state until August 31st, 2015. In Phase two (September 1st, 2015—September 15th, 2015) a 1.49 km section of fence between the western fence-gap and a private property fence was completely removed, including the removal of bollards at the fence-gap (but the stone wall remained), allowing all species unencumbered access between the two conservancies. At this point in time, the old fence-gap wall became a “ghost” gap. We considered animals crossing over the wall or within a few meters of the wall’s northern edge as crossing through the ghost-gap, a linear distance of 30 m. We could not detect movement further south of our camera placement as it pointed north, consequently, our data underestimates crossing numbers by not recording passage south of the wall.

**Figure 6:**
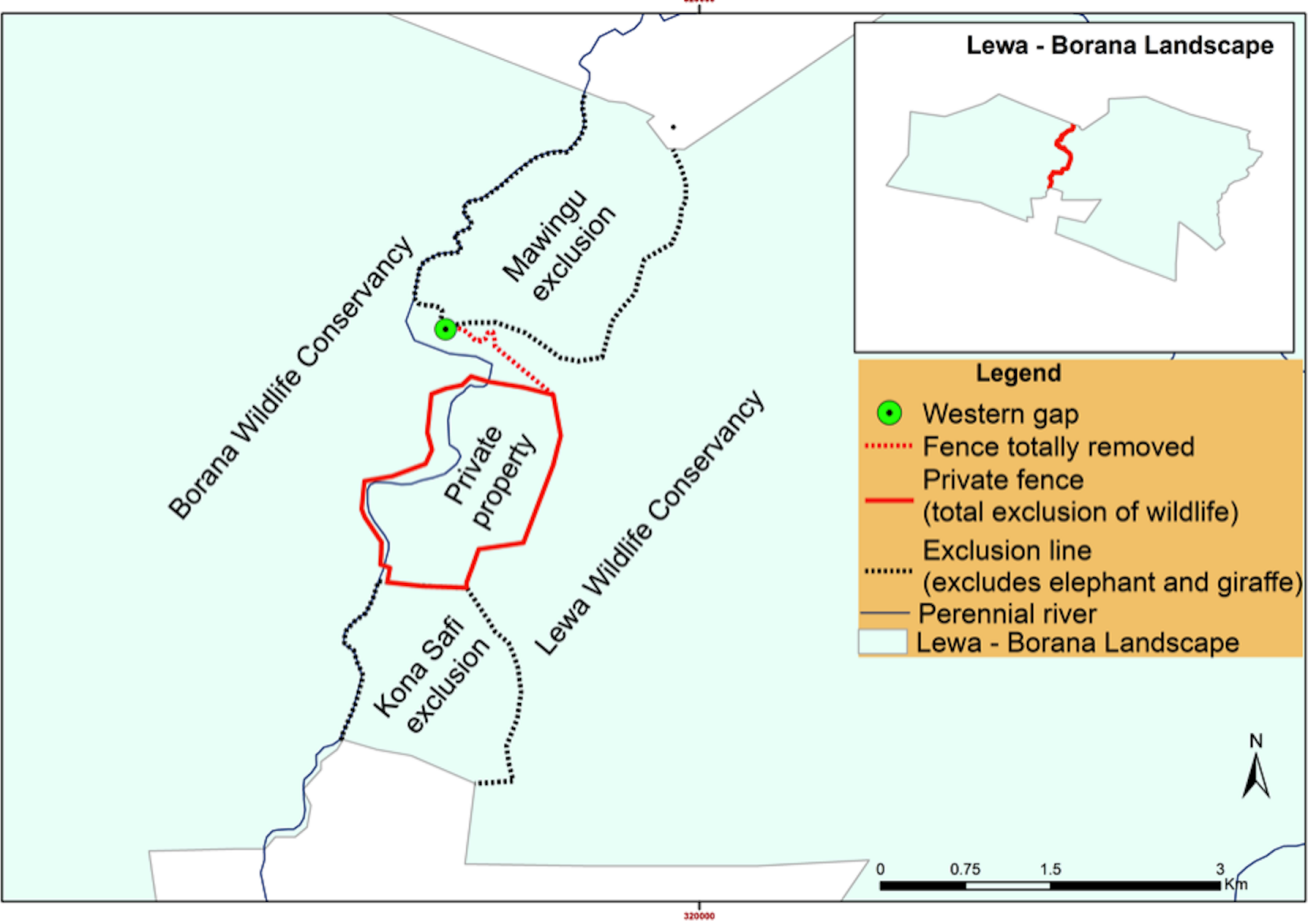
LBL border post-fence removal and modification (after September 2015). Black dotted lines represent two-strand electric exclusionary fencing, solid red line represents 12-strand electrical fence and the dotted red line represents where all fencing was removed.

In the end, management retained a combination of complete fencing (around a private property), exclusionary fencing around areas of sensitive vegetation set aside for black rhino (Mawingu and Kona Safi exclosures) and a section with no fencing at all (Fig. 6) as a semi-porous border between the two conservancies. We continuously monitored the fence-gap over the 2013–2017 period. Because of the potential disruption to wildlife movement caused by the fence reconfiguration, we chose to ignore data pertaining to the 12-month period surrounding the fence removal and to compare a period from before the work had started to after the work had completely finished. We also continued to monitor wildlife traffic at the northern fence-gap as a control site given that this fence-gap had not been altered. Although these two fence gaps are comparable in size and structure, there are many crucial differences between them that make the northern fence-gap a less than ideal control site. The northern fence-gap historically receives greater traffic volume than the western fence-gap and leads to pastoral community lands whereas the western fence-gap leads into a protected wildlife conservancy.

### Prey mortality

Because predation pressures and changes to the predator–prey dynamics can affect prey movement, we collected prey mortality data and determined cause of death (predator kills, illness, drought, electrocution, etc.) for each mortality event in a systematic fashion. We monitored prey preferences of lion by calculating a prey selectivity index before and after fence removal, following Hayward & Kerley (2005) and computing the Jacob’s selectivity index for predator kills (Jacobs, 1974). The index ranges from −1 to +1, where negative values represent relative avoidance and positive values indicate relative preference.

### Aerial monitoring

We also flew a drone equipped with a camera (DJI Phantom 4 Pro, Shenzhen, Guangdong, China) over the ghost-gap location to examine if any new wildlife trails had been formed. We flew the drone on February 22, 2018, more than 30 months after the complete removal of the fence.

### Statistical analysis

We used a non—parametric Chi-Square using SPSS v.24 (IBM Corporation, Armonk, NY, USA) to test for differences in total wildlife usage of the fence-gap, comparing the number of individual animals crossing during a 19-month period pre-fence removal (February 1st, 2013—August 31st, 2014) and a similar 19-month period post-fence removal (February 1st, 2016—August 31st, 2017).

Permits: All necessary permits were obtained for the described field study from the appropriate agencies (Kenya Wildlife Service Affiliation, KWS/BRM/5001, and Kenyan National Council for Science and Technology, NCST/RRA112/I/NIASI).

## Results

We recorded 14 species crossing the fence-gap pre-fence removal versus recording 22 species using the ghost-gap (post-fence removal). Surprisingly, nine of these 22 species had not used the gap in the pre-fence removal period. The individual and group crossings for each species are detailed in Table 1. We compared the number of individuals and groups crossing before and after the fence removal as well as group size. The total number of overall individual animals crossing at the ghost-gap was significantly lower after the fence was removed (Pre = 4,007, Post = 2,457, Chi-Square = 371.7, d*f* = 1, *p* < 0.001). However, the number of wildlife crossing at the northern fence-gap (a control site) was also significantly lower (Pre = 29,764; Post = 20,500, Chi-Square = 1707.4, d*f* = 1, *p* < 0.001), mostly due to a drop in elephant and giraffe traffic over that period.

**Table 1:** Number of wildlife crossings detected between Lewa and Borana. February 1, 2013—August 31, 2014 and February 1, 2016—August 31, 2017. Lewa data.

	Species	Pre individual count number of groups (to Lewa, to Borana)	Post individual count number of groups (to Lewa, to Borana)	Pre group size mean (std) to Lewa, to Borana	Post group size mean (std) to Lewa, to Borana
1	Black rhino (*Diceros bicornis*)	0	26 20 (12, 8)	NA	1.3 (0.6) 1.3 (0.7)
2	Buffalo (*Syncerus caffer*)	0	26 819 (13, 6)		8.8 (9.7) 25.5 (29.7)
3	Bushbuck (*Tragelaphus scriptus*)	2 2 (1, 1)	4 3 (1, 2)	1 1	1 1.5 (0.7)
4	Caracal (*Caracal caracal*)	1 1 (1, 0)	0	1 0	
5	Cheetah (*Acinonyx jubatus*)	1 1 (1, 0)	0	1 0	
6	Eland (*Taurotragus oryx*)	34 28 (14, 14)	94 28 (15, 13)	1.1 (0.3) 1.1 (0.5)	4.1 (3.2) 2.5 (2.1)
7	Elephant (*Loxodonta africana*)	3,071 337 (172, 165)	927 213 (100, 113)	8.4 (9.1) 9.9 (9.3)	4.4 (4.8) 4.2 (4.1)
8	Giraffe (*Giraffa camelopardalis reticulata*)	83 (70)	99 (78)	1.2 (0.4) 1.2 (0.4)	1.3 (0.7) 1.2 (0.5)
9	Grant gazelle (*Nanger granti*)	0	7 4 (2, 2)		2 (1.4) 1.5 (0.7)
11	Grevy’s zebra (*Equus grevyi*)	0	14 10 (6, 4)		1.5 (0.8) 1.25 (0.5)
12	Hartebeest (*Alcelaphus buselaphus cokii*)	0	2 2 (1, 1)		1 1
14	Impala (*Aepyceros melampus*)	2 1 (1, 0)	3 1 (1, 0)	2 0	3 0
15	Jackal (*Canis mesomelas*)	2 1 (1, 0)	2 2 (0, 2)	0 2 (0)	0 1 (0)
10	Kudu (*Tragelaphus strepsiceros*)	0	27 8 (3, 5)		3.7 (2.5) 3.2 (1.3)
16	Leopard (*Panthera pardus*)	14 14 (5, 9)	21 14 (8, 9)	1 (0) 1 (0)	1.3 (0.7) 1.2 (0.4)
17	Lion (*Panthera leo*)	23 14 (7, 7)	58 16 (3, 13)	1.3 (0.5) 1.4 (0.5)	1.3 (0.6) 4.2 (3.6)
18	Mongoose (*Herpestes ichneumon*)	0	2 2 (1, 1)		1 1
19	Ostrich (*Struthio molybdophane*)	0	4 3 (2, 1)		1.5 (0.7) 1
20	Plains zebra (*Equus quagga)*	0	475 102 (50, 52)		4.7 (4.2) 4.6 (3.2)
13	Spotted Hyena (*Crocuta crocuta*)	390 194 (71, 123)	268 159 (79, 80)	2.1 (2.1) 2.0 (1.6)	1.6 (1.6) 1.7 (1.4)
21	Warthog (*Phacochoerus africanus*)	1 1 (1, 0)	9 8 (5, 3)	1	1.2 (0.5) 1 (0)
22	Waterbuck (*Kobus ellipsiprymnus defassa*)	362 283 (150, 133)	103 35 (14, 21)	1.3 (0.7) 1.2 (0.5)	3.1 (2.5) 2.9 (2.9)
23	White Rhino (*Ceratotherium simum*)	0	36 33 (15, 18)		1.1 (0.4) 1.1 (0.2)
24	Wilddog (*Lycaon pictus*)	20 3 (1, 2)	26 4 (3, 1)	9 5.5 (2.1)	8.3 (0.6) 1
	Grand total	4,008	2,475		

**Note:**

Pre: number of individual animals detected crossing at the fence-gap pre-fence removal (number of crossing events). Post: number of individual animals detected crossing at the ghost-gap post fence removal (number of crossing events). Crossings: number of group crossings (groups include individuals crossing alone), and direction (to Lewa and to Borana). Group size: mean and standard deviation of group size.

Individual Crossings: We tested the individual responses of the major species using the western fence-gap before and after the modifications and compared these to the same period at the control site (Table 2). Elephant, waterbuck (*Kobus ellipsiprymnus defassa*), spotted hyena (*Crocuta crocuta*) and giraffe were the main species using the western fence-gap prior to its modification. Post fence removal, we noted that elephant, waterbuck and spotted hyena individual crossings at the ghost-gap had diminished significantly. Giraffe individual crossings were not significantly different. Buffalo (*Syncerus caffer*), plains zebra (*Equus quagga)*, eland (*Taurotragus oryx*) and lion (*Panthera leo*) individual crossings has increased significantly. Of note is that both species of rhinoceroses crossed at the ghost-gap where they had been previously incapable of crossing the original western-gap structure (as it was designed to impede rhinoceros leaving Lewa).

**Table 2:** Comparing wildlife crossing of species most frequently detected at the western fence-gap pre and post fence removal as well as at the northern fence-gap, a control site with no fencing alterations.

Top species	Western pre	Western post	Chi-square test	Northern pre	Northern post	Chi-square test
Elephant	3,071	909	*X*^2^ = 1174.4, *p* < 0.001	9,735	7,550	*X*^2^ = 276.2, *p* < 0.001
Giraffe	83	99	*X*^2^ = 1.4, *p* = 0.236	4,158	2,088	*X*^2^ = 686.0, *p* < 0.001
Hyena	389	268	*X*^2^ = 22.3, *p* < 0.001	1,151	1,448	*X*^2^ = 33.9, *p* < 0.001
Plains zebra	0	475	*X*^2^ = n.a.	13,544	7,748	*X*^2^ = 1577.8, *p* < 0.001
Lion	23	58	*X*^2^ = 15.1, *p* < 0.001	182	173	*X*^2^ = 0.228, *p* = 0.633
Wilddog	20	26	*X*^2^ = 0.783, *p* = 0.376	24	4	*X*^2^ = 14.3, *p* < 0.001
Eland	34	94	*X*^2^ = 28.1, *p* < 0.001	0	0	No test
Waterbuck	362	103	*X*^2^ = 144.3, *p* < 0.001	0	0	No test
Buffalo	0	268	*X*^2^ = n.a.	0	0	No test
Overall	4,008	2,475	*X*^2^ = 371.7, *p* < 0.001	29,764	20,500	*X*^2^ = 1707.4, *p* < 0.001

Group Crossings: We also examined the number of group crossings per species (Table 1), and we found that for elephant both the number of group crossings and the size of the groups had diminished. Also of note was that the number of crossing groups for eland was unchanged, yet the average size of these groups had grown (so more individuals had crossed post fence removal). Waterbuck group sizes had also increased but the number of groups crossing decreased, resulting in fewer individuals crossing the ghost-gap.

Direction of Crossing: In general, we found that species travelled through the fence-gap in both directions more or less equally. The exceptions were buffalo and lion. Buffalo and lion travelled out of Lewa (into Borana) in larger groups.

Control site: We found that elephant traffic had diminished traffic through both the ghost-gap and northern fence-gaps post fence removal. Giraffe traffic at the control site had reduced significantly but not at the ghost-gap. Hyena traffic had increased significantly at the control fence-gap but had diminished significantly at the ghost-gap. Plains zebra numbers had dropped significantly at the control site but significantly increased at the ghost-gap. Notably, buffalo also started using the ghost-gap but not the control site.

Prey Selection: Comparing the results of lion prey selectivity index before and after fence removal suggested that there had been a change in prey preference, notably that buffalo were becoming a more regular staple in the lion diet, that is, their avoidance scale had moved from −0.7 to −0.3 and that plains zebra were slightly more preferred prey than previously, up from 0.3 to 0.4 (Table 3).

**Table 3:** Prey-Selectivity Index on the LBL pre and post fence removal.

Common name	Species	Mean population from census data (2013 and 2014)	Mean population from census data (2016 and 2017)	Lion kills (pre-fence removal)	Lion kills (post-fence removal)	Jacob’s Index (D)—pre-fence removal	Jacob’s Index (D)—post-fence removal
Plains zebra	*Equus quagga*	951	822	70	80	0.3	0.4
Buffalo	*Syncerus caffer*	621	994	6	32	−0.7	−0.3
Grevy’s zebra	*Equus grevyi*	290	293	27	24	0.4	0.2
Giraffe	*Giraffa camelopardalis reticulata*	161	178	16	18	0.4	0.3
Eland	*Tragelaphus (Taurotragus) oryx*	183	154	8	13	−0.1	0.2
Beisa Oryx	*Oryx gazella beisa*	88	138	5	3	0.1	−0.4
Waterbuck	*Kobus ellipsiprynmus defassa*	97	109	7	7	0.2	0.1
Impala	*Aepyceros melampus*	792	829	6	4	−0.8	−0.9
Warthog	*Phacochoerus africanus*	45	51	11	6	0.7	0.4
Total		3,228	3,568	156	187		

**Figure 7:**
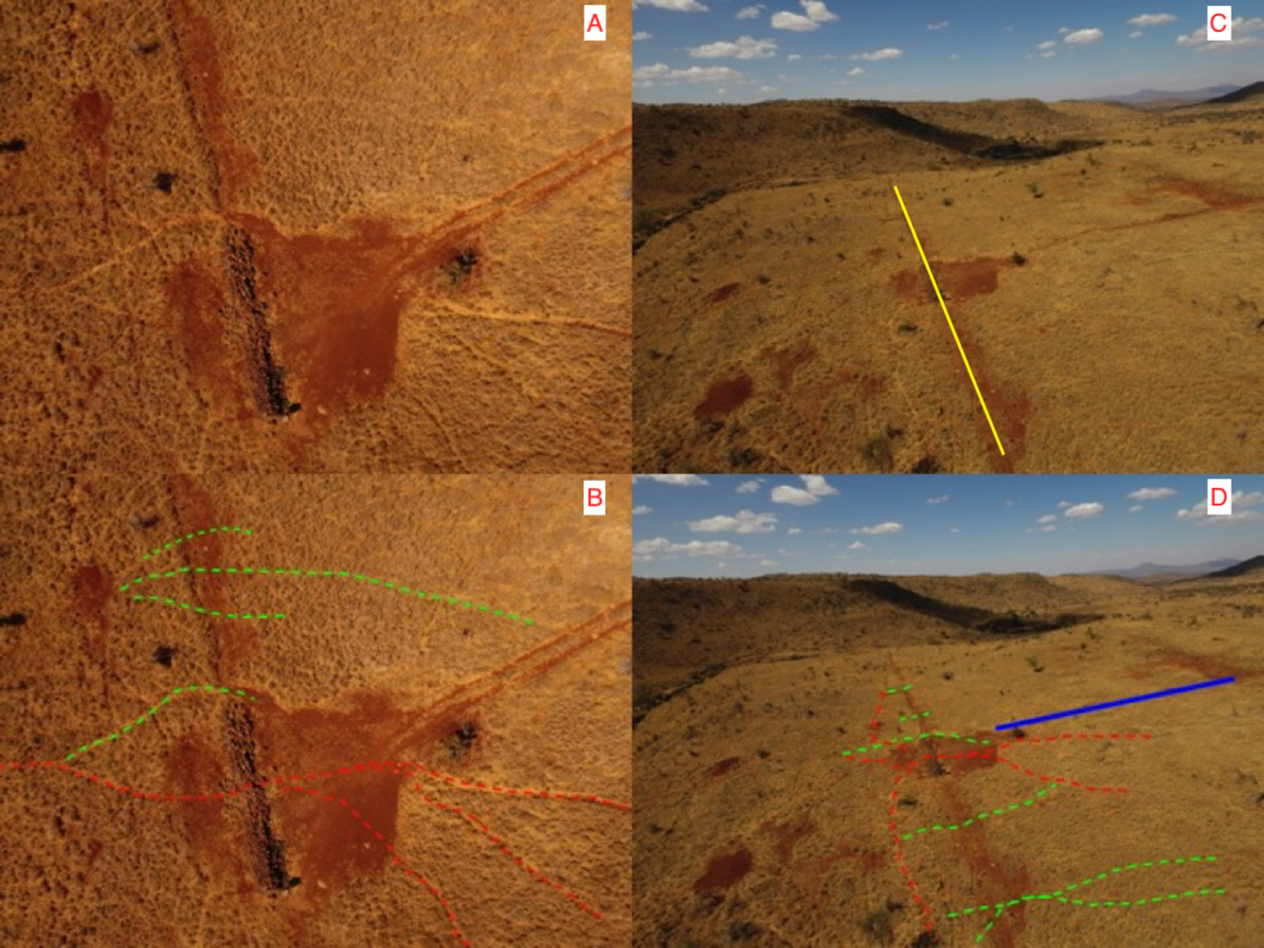
Wildlife paths near the ghost-gap. (Photo credit, Z. Davidson). (A) and (B) show a close-up aerial view directly above the visible rock wall that was the site of the fence-gap connecting the two conservancies prior to the fence removal. Red dashed lines on (B) indicate the main wildlife trails used prior to the perimeter fence being removed. Green dashed lines represent some of the new wildlife trails that cross the old fence line (visible as a linear scar on the landscape and as a yellow line in C) in areas where the fence used to stop all wildlife crossing. (C) and (D) show a more distant view (pointing north) of the same area, dashed lines have similar meaning. The solid blue line in (D) is to indicate the location of the vehicle path visible in both (A) and (B).

Aerial Survey: Aerial photography more than 2 years after the complete removal of the fencing clearly shows the scar of the old fence line and the animals paths used along that fence line converging on the location what was the western fence-gap and rock wall. We noted that many new wildlife paths had been formed that crossed the old fence line (see Fig. 7). Some of these paths were immediately adjacent to the rock wall and our fixed camera-trap (pointing northwards) would have captured wildlife crossing along these proximate paths. Other smaller paths had developed further to the north and to the south (behind our camera-trap), out of the range of the camera trap and thus animals using these could not have been counted.

## Discussion

Increasing connectivity between adjacent conservancies is a laudable goal as it offers more habitat diversity for wildlife and removing fences between two properties should create a more connected habitat. As expected, we did record significant declines in the elephant traffic at the ghost-gap. However, we also recorded a similar absolute drop in elephant numbers crossing at the control site where no fence removal had occurred. Therefore, the reduction of elephant traffic at the ghost-gap might not be solely because of the removal of the fence and might be the result of fewer elephant across the wider landscape. Post-fence removal, elephant and other wildlife using the ghost-gap were observed avoiding the rock wall by crossing immediately adjacent to it (crossing where the fence and the wall connected) and then reconnecting to the main wildlife trail on the other side. Elephant also show detailed spatial knowledge that enables them to minimize distance traveled to nearby watering holes (Polansky, Kilian & Wittemyer, 2015) and thus could have optimized travel distances by selecting different paths across the LBL boundary that avoided the ghost-gap. Our aerial photos show a network of newly created wildlife trails that circumvent the ghost-gap. However, elephants did continue to use the ghost-gap even though they had ample other options available to them. Elephant, because of their great mass, create networks of deeply grooved movement trails that can be used for centuries (Haynes, 2012). Path fidelity can be beneficial during migrations or when negotiating difficult terrain or risky habitat.

After the removal of the fence, spotted hyena and waterbuck, reduced their usage of the fence-gap, yet these species did not completely abandon the gap. Similarly, giraffe, eland, lion, leopard (*Panthera pardus*) and wild dog (*Lycaon pictus*), did not abandon the trail but continued to use or increased their movement through the ghost-gap, demonstrating a surprising level of path fidelity.

Remarkably, nine species that had never used the western fence-gap prior to the removal of the fence began using the ghost-gap. Both species of rhinoceroses had shown interest in the fence-gap before but were unable to cross due to its unique rhino-proof design. Therefore, we were not surprised that rhino might explore the trail once it opened up although rhino had gained access to the complete 8.29 km border after the fence removal and therefore there was no need to cross at the ghost-gap location anymore. This may suggest a high level of spatial awareness of their environment, even to the level of detailed knowledge of obstructions in their movement routes.

The remaining seven species that had never used the western fence-gap prior to the fence being removed were: buffalo, plains zebra (*Equus quagga)*, Grevy’s zebra, greater kudu (*Tragelaphus strepsiceros*), mongoose (*Herpestes ichneumon*), Somali ostrich (*Struthio molybdophane)* and hartebeest (*Alcelaphus buselaphus cokii*). These species, which were able to cross at the western fence-gap before the fence removal, were never observed doing so; yet after the fence was removed, began crossing at the ghost-gap location. What could have caused this phenomenon? Some of these species (kudu, ostrich and hartebeest) have crossed only infrequently and are not abundant at our study site and thus could have taken many years to discover the location of the trail leading through the ghost-gap. However, Grevy’s zebra, plains zebra and buffalo are abundant on the LBL, and it is therefore less probable that these species had not come across the trail leading to the western fence-gap in the past (both zebra species use the northern fence-gap extensively). Once in use, the trails that lead to the fence-gaps collect dung and become easy to find, as the dung of some species such as elephant can last for months, especially in dry seasons (Anderson & Coe, 1974; Barnes & Barnes, 1992; Inogwabini, Mbende & Mbenzo, 2011). Odor is important for encouraging animals to use a “safe” trail; conversely, odor cues can provide information about routes that may be less safe, such as those used by humans. Mammals can smell fear in conspecifics (Ackerl, Atzmueller & Grammer, 2002) and presumably could avoid areas where fear has been experienced by others. Mammals can detect predators through scent and will modify their behavior (Barros et al., 2002; Parsons & Blumstein, 2010; Parsons et al., 2007; Scheinin et al., 2006). Therefore, the scent signal (safe or unsafe) at the fence-gaps will depend on the wildlife stress levels, and these levels will be in part determined by the amount of predator activity in the area. The combination of high historical usage of the wildlife trails passing through the fence-gaps and that predators don’t hunt near the fence-gaps on the LBL (i.e., these pinch-points have not become prey-traps) might have created a safe wildlife movement trail.

Predator disruption could be influencing buffalo and zebra to move out of certain areas and explore new habitat. Given the fact that buffalo and zebra had gained access to the full length of the removed fence line, we find it noteworthy that they only began to use this crossing location after the fence removal. Lions move in and out of the LBL on a regular basis and changes in the competitive landscape can shift hunting preferences (Valeix et al., 2009). In the past, the lion population at our site had preferentially targeted zebra (both species) and avoided buffalo (Dupuis-Desormeaux et al., 2016b), however, it appears that lion were now becoming more interested in buffalo as evidenced by the shift in the Jacob’s selectivity index post fence removal. Coincidently, this shift in diet overlaps with new male lions arriving on the LBL. Consequently, the herds of buffalo that had been growing steadily in numbers (as evidenced in the census data presented in Table 3) on the LBL might have been influenced by predation pressure and begun to seek alternative, less risky habitats to forage within and begun to use the ghost-gap “safe” trail although many alternative options were now available to them.

## Conclusion

What are the implications from a management perspective from these results? The removal of the fence and the resulting obsolescence of a fence-gap have yet to significantly reduce the importance of this area as a pinch-point in the movement landscape. Prey and predator species continue to move through the ghost-gap. However, predator–prey dynamics at our site showed signs of change and these new dynamics might have influenced the use of well-established wildlife trails. We speculate that wildlife continues to use this particular trail through the ghost-gap because it is a relatively safe movement trail. It may well be that more time is required until wildlife abandons this obsolete crossing structure. We did find evidence of a new network of paths crossing the old fence line on both sides of the ghost-gap, suggesting that these changes have already begun. However, it is possible that the wildlife trail that passes through the ghost-gap has become a critical connective link in the landscape for wildlife and that wildlife will keep using this pinch-point until the safety of travel through that location is compromised. This study raises many questions regarding the drivers of wildlife movement and how to increase habitat connectivity between two previously fenced conservancies.

## Supplemental Information

10.7717/peerj.5950/supp-1 Supplemental Information 1 LBL Wildlife Crossing Data- 2013–2017.

10.7717/peerj.5950/supp-2 Supplemental Information 2 Prey mortality data.

## Additional Information and Declarations

### Field Study Permissions

The following information was supplied relating to field study approvals (i.e., approving body and any reference numbers): All necessary permits were obtained for the described field study from the appropriate agencies (Kenya Wildlife Service Affiliation, KWS/BRM/5001, and Kenyan National Council for Science and Technology, NCST/RRA112/I/NIASI).

### Data Availability

The following information was supplied regarding data availability: Raw data is provided in the Supplementary Materials.
